# Sensitisation to mitoxantrone-induced apoptosis by the oncolytic adenovirus Ad∆∆ through Bcl-2-dependent attenuation of autophagy

**DOI:** 10.1038/s41389-017-0020-8

**Published:** 2018-01-24

**Authors:** Carmen Aguirre-Hernández, Héctor Maya-Pineda, Julia San Millán, Y. K. Stella Man, Yong-Jie Lu, Gunnel Halldén

**Affiliations:** Centre for Molecular Oncology, Barts Cancer Institute, Queen Mary University of London, London, UK

## Abstract

Anti-apoptotic Bcl-2 is frequently activated in human malignant cells to promote cell survival and inhibit cell death. Replication-selective oncolytic adenoviruses deleted in the functional Bcl-2 homologue E1B19K potently synergise with apoptosis-inducing chemotherapeutic drugs, including mitoxantrone for prostate cancer. Here, we demonstrate that our previously generated oncolytic mutant Ad∆∆ (E1B19K- and E1ACR2-deleted) caused potent synergistic apoptotic cell death in both drug-sensitive 22Rv1, and drug-insensitive PC3 and PC3M prostate cancer cells. The synergistic cell killing was dependent on Bcl-2 expression and was prevented by Bcl-2 knockdown, which led to activation of the autophagy pathway. Mitoxantrone-induced autophagy, which was decreased in combination with Ad∆∆-infection resulting in increased apoptosis. Expression of the viral E1A12S protein alone mimicked the synergistic effects with Ad∆∆ in combination with mitoxantrone while intact wild-type virus (Ad5) had no effect. Early and late-stage inhibition of autophagy by Atg7 knockdown and chloroquine respectively, promoted apoptotic cell killing with mitoxantrone similar to Ad∆∆. These findings revealed currently unexplored actions of E1B19K-deleted oncolytic adenoviruses and the central role of Bcl-2 in the synergistic cell killing. This study suggests that cancers with functional Bcl-2 expression may be selectively re-sensitised to drugs by Ad∆∆.

## Introduction

Clinical safety and promising anti-tumour efficacy has been demonstrated for oncolytic adenoviral mutants targeting solid cancers, with significant tumour-regression in combination with cytotoxic drugs or radiation therapy, for example, the oncolytic mutants CG7870 and Ad5-yCD/mutTK(SR39)rep-ADP^1,2^. Currently adenoviral mutants with deletions in the viral E1ACR2-region are the most promising clinical candidates with high potency and selectivity, such as *dl*922-947 and Ad∆24^3–6^. The E1ACR2 domain in the early viral E1A gene binds to pRb that inactivates the G1/S checkpoint and induces S-phase, which is essential for viral propagation in normal cells but not in cancer cells with deregulated cell cycle. We developed the novel mutant Ad∆∆ with the anti-apoptotic E1B19K gene deleted in addition to the E1ACR2-deletion^7,8^. E1B19K is a functional Bcl-2 homologue and plays a dual role in apoptosis and autophagy. It binds to Bax/Bak inhibiting mitochondrial pore formation and apoptosis in response to death receptor-activation and intrinsically induced apoptosis (p53-dependent and -independent)^9^. E1B19K also interacts with Beclin-1 disrupting the Bcl-2/Beclin-1 complex that may promote autophagy^10^. We previously demonstrated that viruses with the E1B19K-deletion greatly enhanced cell killing induced by DNA-damaging agents in pancreatic cancer models^7,11^. The Ad∆∆ mutant efficiently eliminates prostate and pancreatic cancers in preclinical models and augments potency of current clinical cytotoxic drugs including mitoxantrone, docetaxel, gemcitabine and irinotecan, even in drug-insensitive cells and in murine xenograft models^7,8,11–13^. We demonstrated that expression of the early viral E1A12S protein alone was sufficient for the synergistic interactions with these drugs, in agreement with previous studies^11,14–16^. The exact mechanisms involved in this sensitisation have not been clearly delineated and depend on cell type and specific gene-alterations^14,17^. The small E1A12S protein is one of two major splice variants of the essential viral *E1A* gene which, in the absence of E1B19K induces apoptosis but not viral replication, in contrast to the second major splice product E1A13S. We used the non-replicating viral vector AdE1A12S to investigate the role of E1A in drug-sensitisation in the absence of other viral proteins and replication.

In the current study, using prostate cancer as a model we investigated cellular pathways that are involved in virus-mediated sensitisation to mitoxantrone. In particular, the sensitisation to apoptosis, aiming to identify mechanisms that are utilised by E1B19K-deleted mutants to overcome treatment-resistance allowing for future development of improved therapies. Prostate cancer is the second most common cause of cancer-related deaths in men in Western countries^18^. Although the initial response to anti-androgens is good, resistance unavoidably develops to all current therapeutics. The cytotoxic drugs mitoxantrone and docetaxel are frequently administered but have only palliative effects while novel targeted therapies such as abiraterone may be more efficacious in some patients^19^. We and other investigators have demonstrated that a different strategy, using replication-selective oncolytic adenoviruses, can selectively and potently reduce growth and progression of therapy-resistant prostate cancer in pre-clinical models^4,8,20^. Because of the central role for Bcl-2 in preventing both apoptosis and autophagy, we investigated its role in virus-mediated sensitisation to mitoxantrone. We used the androgen-independent PC3 and PC3M, and the androgen-sensitive 22Rv1 human prostate cancer cells^4,15^. PC3 and PC3M cells are metastatic prostate cancer models, which are highly insensitive to drugs. It was previously reported that therapeutics currently used to treat prostate cancer activated cellular autophagy, resulting in poor treatment-responses and development of resistance, including to bicalutamide^21^, enzalutamide^22^, taxanes^23^ and radiotherapy^24^. We hypothesised that the resistance to mitoxantrone involved activation of cell survival mechanisms that could be subdued by viruses to increase cell killing, and autophagy may be such a mechanism.

Inactivation of the autophagy suppressive Bcl-2/Beclin-1 complex by Bcl-2 knockdown, potently induced autophagy and ablated Ad∆∆ induced sensitisation to mitoxantrone. In PC3, 22Rv1 and PC3M cells, Ad∆∆ promoted mitoxantrone-induced apoptosis and reduced mitoxantrone-activated autophagy that was dependent on Bcl-2 expression. The importance of autophagy attenuation and apoptosis induction was confirmed using the late-stage pharmacological inhibitor chloroquine and knockdown of Atg7 that prevented autophagy initiation. Our data revealed cellular mechanisms that may be further exploited for developing improved therapies for prostate cancer patients by retaining the Bcl-2/Beclin-1 complex for autophagy-inhibition.

## Results

### The adenoviral mutants Ad∆∆ and AdE1A12S synergistically enhance mitoxantrone-induced apoptosis in human prostate cancer cell lines

**Fig. 1:**
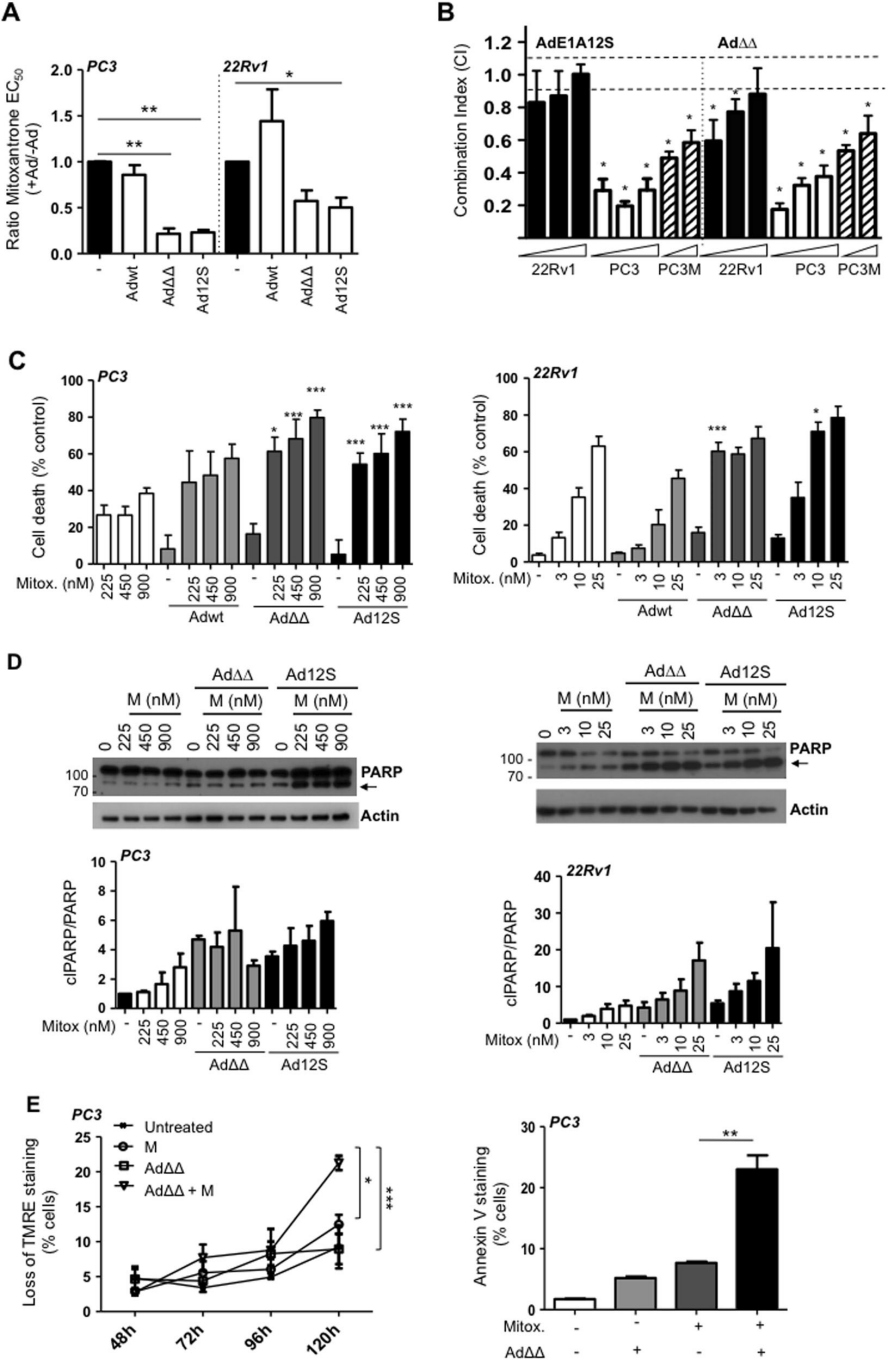
AdΔΔ and AdE1A12S synergise with mitoxantrone to induce apoptotic cell killing in prostate cancer cells. **a** Sensitisation to mitoxantrone by Ad5wt, Ad∆∆ and AdE1A12S in PC3 and 22Rv1 cells. Dose−response curves to mitoxantrone with and without fixed doses of virus killing 10–40% of cells alone; Ad5wt or AdΔΔ (1000 ppc; PC3 and 25 ppc;22Rv1) or AdE1A12S (5000 ppc;PC3and 100 ppc;22Rv1). Cell viability determined by MTS assays 5 (PC3) or 3 days (22Rv1) after treatment and data analysed by unpaired *t*-test, averages ± SEM, *n* ≥ 3, **p < 0.01, *p < 0.05. Ratios = EC_50_-values of combination/EC_50_-values mitoxantrone. **b** PC3 and 22Rv1 cells infected with Ad∆∆ or AdE1A12S and treated with mitoxantrone for 5 and 3 days, respectively, at three constant ratios (0.5, 2.5 and 12.5 ppc/nm; indicated by wedges) and PC3M cells at two constant ratios (2.5 and 12.5 ppc/nm). Combination indexes (CI) calculated from isobolograms and synergistic cell killing defined as CI ≤ 0.9; averages ± SD, *n* = 3, *p < 0.05 compared to the theoretical additive values (dashed lines; additive effects 0.9 < CI < 1.1). **c** PC3 cells (left panel) treated with mitoxantrone (225, 450 and 900 nm) and/or infected with Adwt and AdΔΔ at 500ppc or Ad12S at 5000ppc. Cell death indirectly determined using the MTS viability assay 5 days after treatment. One-way ANOVA with Tukey−Kramer post-test, averages ± SEM, *n* = 4. 22Rv1 cells (right panel) treated with mitoxantrone (3, 10 and 25 nm) and/or infected with Ad5wt and AdΔΔ at 15 ppc or Ad12S at 50 ppc. Number of dead cells determined by the Trypan blue exclusion assay after 3 days. Representative study, one-way ANOVA with Tukey−Kramer post-test, averages ± SEM from quadruplicate samples. *p < 0.05, ***p < 0.001, ****p < 0.0001, *compared to the theoretical additive value of mitoxantrone and virus, *n* ≥ 3. **d** Expression of PARP detected by immunoblotting (cleaved PARP indicated with black arrows; 89 kDa), one representative immunoblot, *n* = 3. PC3 cells (left panel) infected with AdΔΔ (500 ppc) or AdE1A12S (5000 ppc) and/or mitoxantrone (225, 450 or 900 nm). 22Rv1 cells (right panel) infected with AdΔΔ (15 ppc) or AdE1A12S (50 ppc) and/or mitoxantrone (3, 10 or 25 nm). Cells were lysed 48 h after treatment. Lower panels: PARP ratios; cleaved PARP (clPARP/PARP) after quantification by densitometry and normalised to the actin loading control, expressed as fold-change relative to the untreated control, *n* = 3. Molecular weight markers indicated in kDa. **e** PC3 cells analysed at the indicated time points for mitochondrial depolarisation by loss of TMRE staining using flow cytometry (left panel) and Annexin V staining (right panel) 120 h after infection with AdΔΔ (500 ppc) and/or treated with mitoxantrone (450 nm). One-way ANOVA with Tukey−Kramer post-test *p < 0.05, **p < 0.01, ***p < 0.001, *n* = 3

We explored whether suboptimal doses (<EC_50_-values) of the replication-selective Ad∆∆ mutant could enhance mitoxantrone-induced cell killing. Both AdΔΔ and the non-replicating AdE1A12S (expressing only E1A12S) decreased mitoxantrone EC_50_-values in the androgen-insensitive PC3 and -sensitive 22Rv1 cells while the intact Ad5wt virus did not sensitise the cells (Fig. 1a). The increased cell killing was synergistic with combination indexes (CI) < 0.9, which was significant with both mutants (*p* < 0.05) in PC3 and PC3M cells (Fig. 1b). Combining suboptimal doses of viruses and drug caused significantly higher levels of cell death than the predicted additive responses in both cell lines, with Ad∆∆ and AdE1A12S but not with Ad5wt (*p* < 0.05; Fig. 1c). Both Ad∆∆ and AdE1A12S sensitised the more aggressive PC3 subline, PC3M cells to mitoxantrone (Supplementary Fig. 1A). The lesser degree of synergy in response to the combinations in 22Rv1 cells might be due to the higher sensitivity to mitoxantrone (>7-fold) in these cells compared to the more insensitive PC3 and PC3M cells (Supplementary Table 1). The increased cell killing was paralleled by PARP cleavage in both PC3 and 22Rv1 cells (Fig. 1d). To investigate whether cell killing was apoptotic under the synergistic conditions, mitochondrial membrane depolarisation was determined over time. In PC3 cells, Ad∆∆ significantly increased mitoxantrone-induced apoptosis after 120 h (*p* < 0.05, 450 nm; Fig. 1e, left panel) with a similar trend in 22Rv1 (10 nm; Supplementary Fig. 1B) and in PC3M cells (*p* < 0.001, 450 and 900 nm; Supplementary Fig. 1C). Increased apoptotic death was verified by significantly higher levels of Annexin V staining in PC3 cells (*p* < 0.01; Fig. 1e, right panel). Virus-infection alone did not cause apoptosis under these conditions. Importantly, viral replication appeared to play a minor role in the synergistic cell killing since both the non-replicating AdE1A12S and the Ad∆∆ mutants sensitised the cells. Importantly, E1A-expression was detected 24 and 48 h after infection with both mutants and was maintained in the presence of mitoxantrone (PC3 cells; Supplementary Fig. 1D).

### Bcl-2 expression is required for Ad∆∆-mediated sensitisation to mitoxantrone

**Fig. 2:**
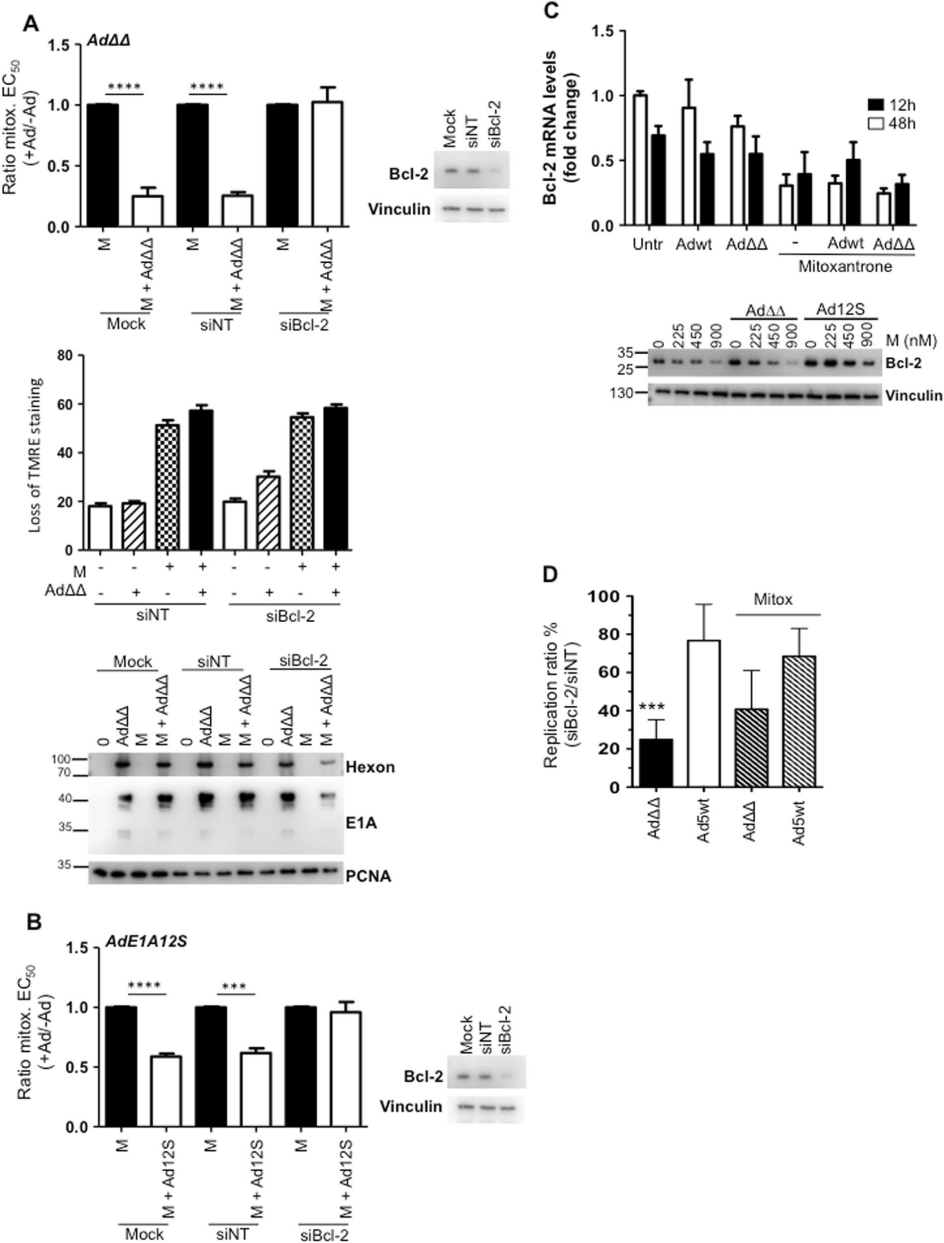
Bcl-2 knock-down prevents AdΔΔ-mediated sensitisation to mitoxantrone in PC3 cells. **a** Upper panel: EC_50_-values generated from dose−response curves to mitoxantrone alone or in combination with AdΔ∆-infection (500 ppc) in cells previously transfected with siRNA or mock transfection (Dharmafect reagent). Ratios of EC_50_-values mitoxantrone+virus/mitoxantrone alone, unpaired *t*-test, **p < 0.01 ****p < 0.0001, *n* = 3. Insert: representative immunoblot verifying Bcl-2 knockdown in each viability study, *n* ≥ 3. Middle panel: Mitochondrial depolarisation as an indicator of apoptosis (loss of TMRE staining) in siRNA-transfected cells and infected with AdΔΔ (500 ppc) ± mitoxantrone (100 nm), analysed 5 days after treatment, *n* = 3. Lower panel; Infection with AdΔΔ (500 ppc) and/or treatment with mitoxantrone (450 nm), in cells previously (4 days) transfected with siRNAs or mock treated, 48 h after treatment and immunoblotted for hexon and E1A, representative blot, *n* = 3. **b** EC_50_-values generated from dose−response curves to mitoxantrone alone or in combination with AdE1A12S-infection (5000 ppc) in cells previously transfected with siRNA as described in **a**, *n* ≥ 3. **c** Infection with Ad5wt or AdΔΔ (500 ppc) and/or treated with mitoxantrone (450 nm) and analysed after 12 and 48 h for changes in Bcl-2 mRNA levels by RT-qPCR (upper panel) and immunoblotting at three doses of drug (lower panels), *n* = 3. **d** Viral replication determined by TCID_50_ in siBcl-2 and siNT transfected cells, expressed as percentages of replication in siBcl-2 cells vs. siNT cells. Cells were infected with Ad5wt or AdΔΔ (100 ppc) and treated with mitoxantrone (M; 100 nm) for 72 h. One-way ANOVA with Tukey−Kramer post-test, ***p < 0.001, *n* = 4

Our findings that mutants deleted in the anti-apoptotic Bcl-2 functional homologue E1B19K, but not wild-type virus, sensitised cells to mitoxantrone, led us to investigate whether Bcl-2 was essential for sensitisation. Transfection of PC3 cells with a Bcl-2 siRNA pool resulted in knockdown of Bcl-2 (Fig. 2a; immunoblot). Interestingly, the absence of Bcl-2 expression precluded both Ad∆∆-mediated sensitisation to mitoxantrone and enhancement of apoptotic death (Fig. 2a; upper and middle panels). In addition, the viral proteins E1A and hexon showed a trend towards decreased expression in the presence of mitoxantrone in the knockdown cells (Fig. 2a; lower panel). To explore whether the prevention of sensitisation was caused by the decreased expression-levels of E1A, the cells were infected with the AdE1A12S mutant that constitutively expresses E1A12S. However, sensitisation was prevented even in AdE1A12S-infected knockdown cells, while it was significant in siNT-transfected and mock-treated cells (*p* < 0.001; Fig. 2b). Notably, siBcl-2-transfected cells had two-fold higher levels of basal cell death than the corresponding siNT cells (Supplementary Fig. 2A, B). In conclusion, these findings demonstrate that Bcl-2 expression was required for AdΔΔ-mediated sensitisation to mitoxantrone.

### The viral E1A12S protein stabilises Bcl-2 expression while mitoxantrone reduces the levels

The requirement for Bcl-2 expression in Ad∆∆-mediated sensitisation led us to investigate whether mitoxantrone or the viral mutants altered the Bcl-2 levels in PC3 cells. Mitoxantrone potently reduced Bcl-2 mRNA levels from 12 to 48 h which was paralleled by dose-dependent decreases in Bcl-2 protein levels (Fig. 2c). Neither Ad5wt nor Ad∆∆ affected Bcl-2 mRNA levels. Notably, infection with the AdE1A12S mutant strongly counteracted the mitoxantrone-dependent decreases in Bcl-2 protein levels with a similar trend in Ad∆∆-infected cells at the lowest drug dose (225 nm) (Fig. 2c; lower panel). These findings suggest that expression of E1A in the absence of E1B19K was sufficient to stabilise Bcl-2. Furthermore, in Bcl-2 knockdown PC3 cells, Ad∆∆ replication was decreased compared to the siNT control cells both in the absence (*p* < 0.001) and presence of mitoxantrone (Fig. 2d). Importantly, Ad5wt replication was less affected by Bcl-2 knockdown and progressed at two-fold higher levels than Ad∆∆. These findings point towards an essential role for Bcl-2 in sensitisation to apoptosis-inducing drugs with E1B19K-deleted oncolytic mutants. The frequent upregulation of Bcl-2 function in prostate cancer ensures the efficient propagation of adenoviral mutants deleted in the E1B19K gene^25^.

### Ad∆∆ and AdE1A12S attenuate mitoxantrone-activated autophagy in the presence of Bcl-2 expression

**Fig. 3:**
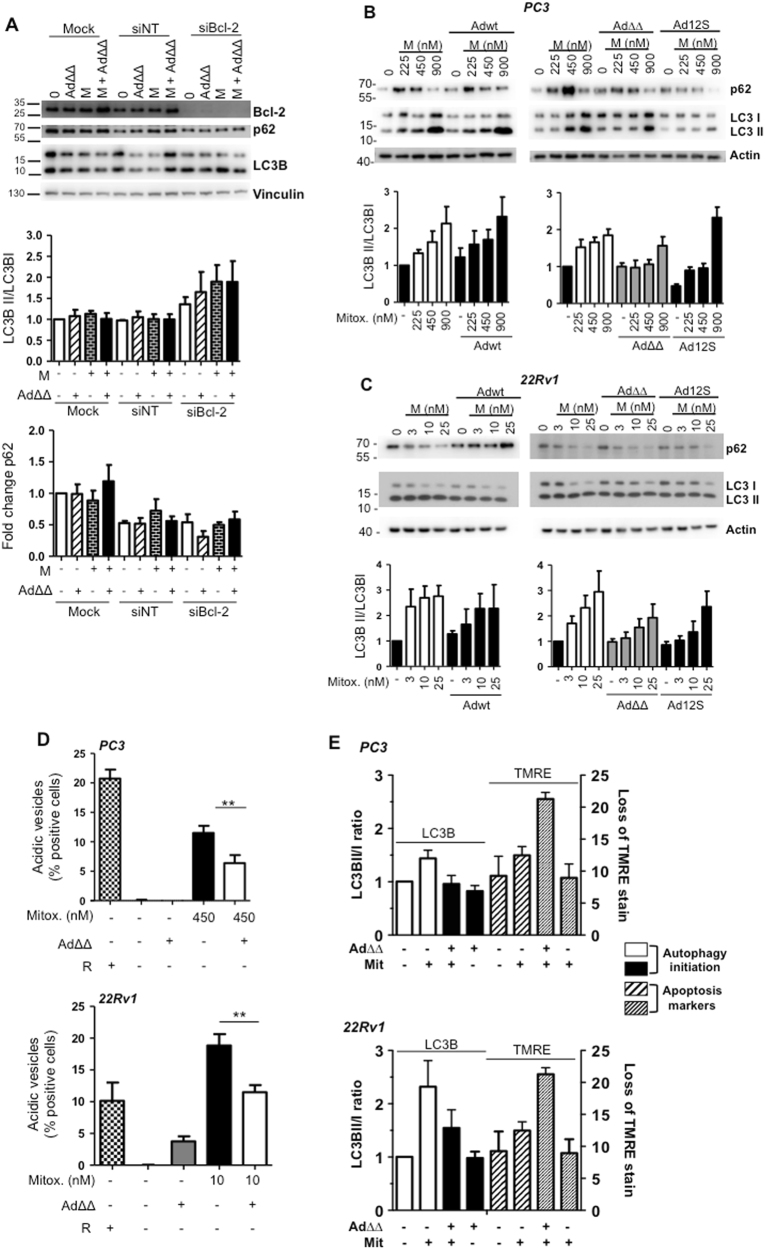
AdΔΔ or AdE1A12S attenuate mitoxantrone-induced autophagy that is dependent on Bcl-2 expression. **a** Infection of PC3 cells with AdΔΔ (500 ppc) and/or treatment with mitoxantrone (450 nm), 4 days after transfection with non-targeting siRNA (siNT) or targeting siRNA (siBcl-2) or mock transfection (Dharmafect reagent) 48 h after treatment and immunoblotted for Bcl-2, p62, LC3B, representative blot. LC3BII/I ratios quantified by densitometry analysis, normalised to the loading control and expressed as fold-change relative to the untreated control, *n* = 3. **b**,** c** Immunoblotting for p62 and LC3BII/I proteins, **b** PC3 cells treated with mitoxantrone (M; 225, 450, 900 nm) and/or infected with Ad5wt or AdΔΔ (500 ppc) and AdE1A12S (5000 ppc), and **c** 22Rv1 cells treated with mitoxantrone (3, 10, 25 nm) and/or infected with Ad5wt or AdΔΔ (20 ppc) and AdE1A12S (100 ppc), representative blots, quantification as in **a**, *n* = 3–6. **d** Lysotracker staining to detect acidic vesicles using flow cytometry. PC3 cells treated with mitoxantrone (450 nm), AdΔΔ (500 ppc) or in combination for 120 h, and 22Rv1 cells treated with mitoxantrone (10 nm) or AdΔΔ (20 ppc) or in combination for 72 h. One-way ANOVA with Tukey−Kramer post-test, **p < 0.01, *n* = 4. Rapamycin (50 nm) was used as a positive control. **e** LC3II/I ratios from immunoblotting data and apoptosis determined by loss of TMRE staining (120 h; PC3 and 72 h; 22Rv1). In all assays cells were treated with mitoxantrone at 10 nm (22Rv1), at 450 nm (PC3) and/or infected with AdΔΔ (20 ppc; 22Rv1 and 500 ppc; PC3), *n* = 3–6

We explored whether the Bcl-2-dependent Ad∆∆-mediated sensitisation to mitoxantrone involved effects on the autophagy pathway in addition to apoptosis-induction^26^. In a previous report, the viral E1B19K protein was demonstrated to displace Bcl-2 from Beclin-1 resulting in autophagy initiation^10^. Autophagy-activation was confirmed in the PC3 Bcl-2 knockdown cells, determined by increased conversion of LC3BI to LC3BII (1.5–2-fold) and decreased p62 expression (Fig. 3a). The increases in LC3BII/I ratios were not reduced by Ad∆∆-infection and the virus did not attenuate mitoxantrone-induced autophagy in the Bcl-2 knockdown PC3 cells. Thus, the absence of sensitisation (Fig. 2a, b) was likely the result of the potent induction of autophagy by release of the Beclin-1/Vps34/Vps35 complex in the absence of Bcl-2 binding.

Importantly, mitoxantrone-activated autophagy, seen as increased LC3BII/I ratios and p62 degradation, markers for autophagy-initiation and -completion, respectively^27^. Mitoxantrone caused 2–3-fold dose-dependent increases in LC3BII/I ratios in PC3, 22Rv1 and PC3M cells (Fig. 3b, c and Supplementary Fig. 3A). In PC3 cells, autophagy did not appear complete at the lower doses of 225 and 450 nm, indicated by higher p62 levels, despite the potent induction of LC3II/I ratios (Fig. 3b). However, slightly decreased p62 expression at all concentrations was noted in 22Rv1 and PC3M cells, suggesting completion of autophagy (Fig. 3b, c and Supplementary Fig. 3A, B). The activation of autophagy in response to suboptimal doses of mitoxantrone is likely to serve as a survival mechanism as has been demonstrated for other cytotoxic drugs^28^. In contrast to the induction of autophagy in Bcl-2 knockdown cells, mitoxantrone-induced autophagy was attenuated by Ad∆∆ and AdE1A12S in PC3 cells and restored basal levels of LC3BII/I ratios at the lower 225 and 450 nm doses but not at 900 nm (Fig. 3b). Similar findings were observed in 22Rv1 and PC3M cells, while no consistent changes in p62 levels were detected with the combinations (Fig. 3b, c). Importantly, Ad5wt had no effect on mitoxantrone-induced autophagy and did not attenuate the conversion to LC3BII. In agreement with the increased LC3II/I ratios in mitoxantrone-treated cells, the number of acidic vesicles was significantly reduced in the presence of AdΔΔ in both PC3 and 22Rv1 cells (*p* < 0.01; Fig. 3d). Importantly, infection with viruses alone had no effect on the autophagy markers, demonstrating that basal autophagy was not altered by adenovirus in these cells.

Taken together, Ad∆∆ efficiently counteracted autophagy-initiation and promoted apoptosis at the lower concentrations of mitoxantrone (225–450 nm; PC3 and 3–10 nm; 22Rv1), with a lesser degree of autophagy-induction. Under these conditions the decreased LC3BII/I ratios correlated with increased mitochondrial depolarisation, resulting in overall improved cell killing in all three cell lines (Fig. 3e, Supplementary Fig. 3B). The correlation indicated that Ad∆∆, through a Bcl-2 dependent mechanism, inhibited autophagosome-formation, directing mitoxantrone-treated cells towards higher levels of apoptosis.

### Chloroquine enhances mitoxantrone-dependent apoptotic cell killing similar to Ad∆∆-infection

**Fig. 4:**
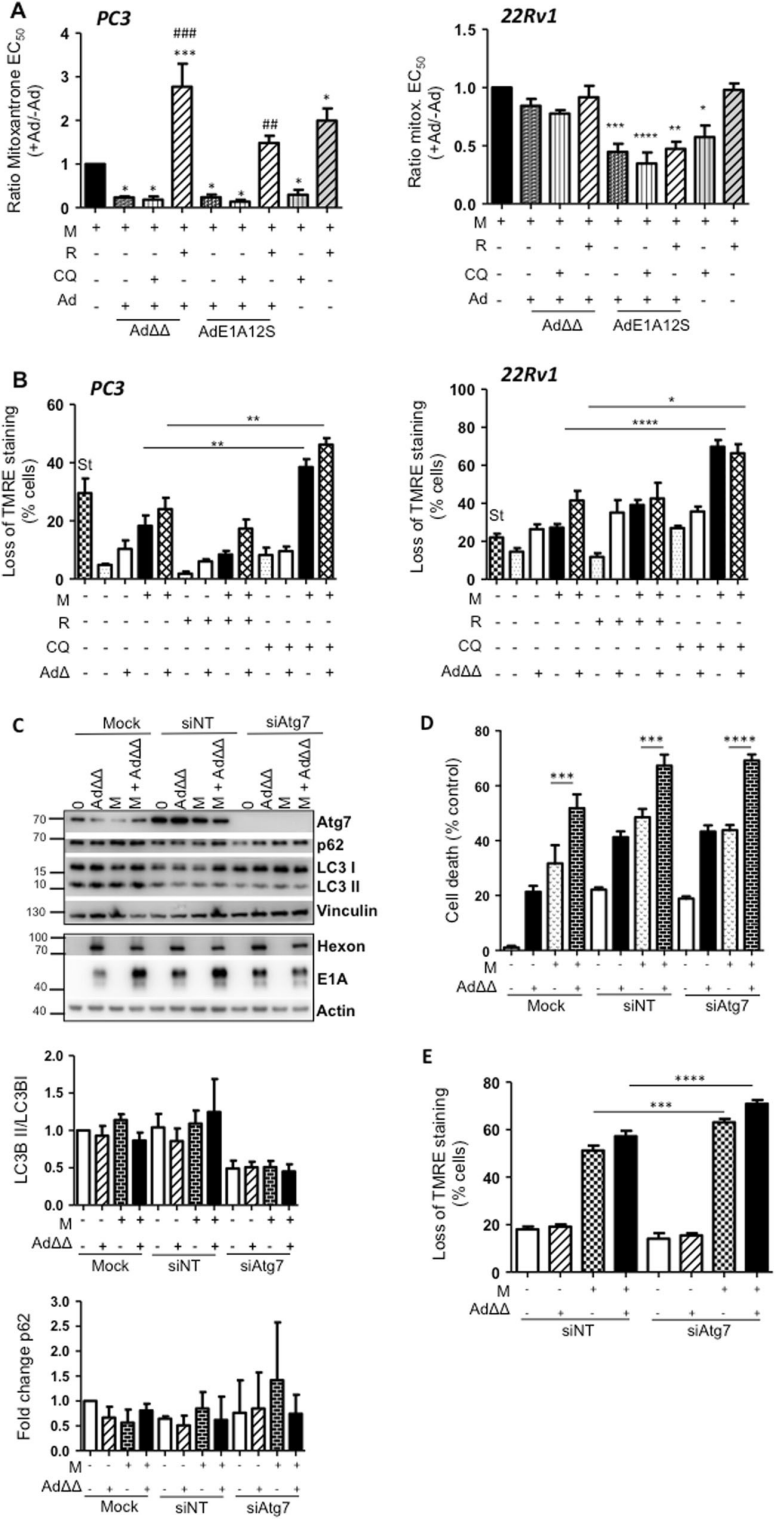
Inhibition of autophagy by chloroquine and Atg7 siRNA enhances mitoxantrone-dependent apoptotic cell killing both in the presence and absence of Ad∆∆. **a** Cell viability determined 5 days after treatment by generating EC_50_-values for mitoxantrone (M) in combination with fixed doses of chloroquine (CQ; 10 μm), rapamycin (R; 50 nm), AdΔΔ (PC3;500 ppc and 22Rv1; 20 ppc) or AdE1A12S (PC3;5000 ppc and 22Rv1;50 ppc), or the combined treatment of CQ or R and one of the viruses. EC_50_-ratios generated from EC_50_-values for M ± virus ± R or ±CQ/EC_50_-value for M alone. Averages ± SEM, *compared to mitoxantrone and #compared to M+virus, *n* ≥ 3. **b** Mitochondrial depolarisation determined in the presence of AdΔΔ (PC3;750 ppc and 22Rv1;20 ppc) and/or mitoxantrone (PC3;450 nm and 22Rv1;10 nm) and/or rapamycin (50 nm), and/or CQ (10 μm) and analysed by TMRE and flow cytometry after 120 h (PC3) and 72 h (22Rv1), St Staurosporin. One-way ANOVA with Tukey−Kramer post-test, *p < 0.05, **p < 0.01, ***p < 0.001, *n* = 4. **c** Infection of PC3 cells with AdΔΔ (500 ppc) and/or treatment with mitoxantrone (450 nm), 4 days after transfection with non-targeting siRNA (siNT) or targeting siRNA (siAtg7) or mock transfection (Dharmafect reagent). Cell lysates collected 48 h after treatment and immunoblotted for Atg7, p62, LC3B, hexon and E1A, representative blot. Lower panels: densitometer quantification of LC3BII/I and p62, *n* ≥ 3. **d** Percent cell death (MTS assay) in PC3 cells previously transfected with the siRNAs and treated with mitoxantrone (60 nm), AdΔΔ (500 ppc) and the combination of both. One-way ANOVA with Tukey−Kramer post-test, ***p < 0.001, ****p < 0.0001, *n* = 4. **e** Mitochondrial depolarisation (loss of TMRE staining) in PC3 cells infected with AdΔΔ (500 ppc) and/or treated with mitoxantrone (100 nm) 24 h after transfection and analysed 5 days after treatment, one-way ANOVA with Bonferroni post-test, ***p < 0.001, ****p < 0.0001, *n* = 3

To delineate the contribution of the autophagy pathway to the enhanced apoptotic cell killing, the effects of the mTOR-inhibitor rapamycin (autophagy-inducer) and the late-stage autophagy-inhibitor chloroquine were investigated. As expected, rapamycin increased LC3II/I ratios and promoted p62-degradation while chloroquine increased LC3BII/I ratios and increased p62 accumulation over time in both PC3 and 22Rv1 cells (24–72 h; Supplementary Fig. 4A, B). In both cell lines, chloroquine greatly decreased mitoxantrone EC_50_-values that were maintained at the lower values when co-infected with AdΔΔ or AdE1A12S (Fig. 4a). In contrast, rapamycin potently desensitised the cells both in the presence or absence of Ad∆∆ and to a lesser degree with AdE1A12S. Furthermore, chloroquine significantly increased mitoxantrone-dependent mitochondrial depolarisation in the presence of Ad∆∆ (2–3-fold) while rapamycin decreased the depolarisation (Fig. 4b). These findings suggest that late-stage autophagy-inhibition enhanced drug-induced cell death by promoting apoptosis similar to Ad∆∆, while autophagy-induction desensitised the cells and promoted survival as observed in the Bcl-2 knockdown cells (Figs. 2a, b and 3e).

To rule out effects on the viral life-cycle by the pharmacological inhibitors which target major protein synthesis and degradation pathways, we investigated potential effects on the viral life cycle. In contrast to the attenuated replication in Bcl-2 knockdown PC3 cells (Fig. 2d), neither rapamycin nor chloroquine had significant effects on viral infection, E1A expression or replication in combination with mitoxantrone (Supplementary Fig. 4C−E) and consequently, the desensitisation and sensitisation were not due to changes in viral propagation but rather direct effects on cellular factors. In contrast, mitoxantrone increased viral infection while replication was reduced, as we previously reported for Ad5 mutants^4^. In Ad∆∆-infected PC3 cells treated with mitoxantrone and chloroquine, E1A expression and viral replication were slightly reduced similar to the observations in Bcl-2 knockdown cells (Fig. 2a, d and Supplementary Fig. 4D, E).

### Ad∆∆ enhances mitoxantrone-dependent apoptotic cell killing in Atg7 knockdown PC3 cells

Elimination of Atg7 has been established to prevent the early stages of autophagosome formation and to be integral for autophagy initiation^27,29^. To investigate whether the pro-apoptotic effects of late-stage autophagy-inhibition by chloroquine also occurred when autophagy was inhibited at an early stage, Atg7 was knocked-down to undetectable levels by specific siRNA pools (Fig. 4c). LC3BI to LC3BII conversion was prevented in these cells even in the presence of mitoxantrone.

Interestingly, Ad∆∆-infection sensitised the siAtg7 knockdown cells to mitoxantrone to the same extent as in mock-treated or non-targeting siRNA (siNT) transfected cells (*p* < 0.01; Fig. 4d, *p* < 0.05; Supplementary Fig. 5). Mitoxantrone-dependent mitochondrial depolarisation was significantly increased in siAtg-7 knockdown cells compared to siNT-treated cells, both in the presence and absence of Ad∆∆-infection (*p* < 0.001; Fig. 4e). These findings demonstrate that apoptosis was promoted when autophagy was inhibited and is in agreement with the increased apoptosis in chloroquine-treated cells (Fig. 4b). Although, in contrast to the chloroquine data, at these early time-points the increased apoptosis was not paralleled by detectable increases in cell death. Importantly, Ad∆∆-infection sensitised cells even under conditions of autophagy-inhibition, demonstrating that active autophagy was not required and that inhibition at any stage was beneficial for Ad∆∆-mediated enhancement of mitoxantrone-induced apoptosis. These findings led us to conclude that Ad∆∆, through potent E1A-expression, reinforced the mitoxantrone-dependent mitochondrial depolarisation and indirectly maintained Bcl-2 bound to the autophagy inhibitory Bcl-2/Beclin-1/Vps34/Vps35 complex.

### LC3BII expression is undetectable in infected cells expressing the viral E1A protein

**Fig. 5:**
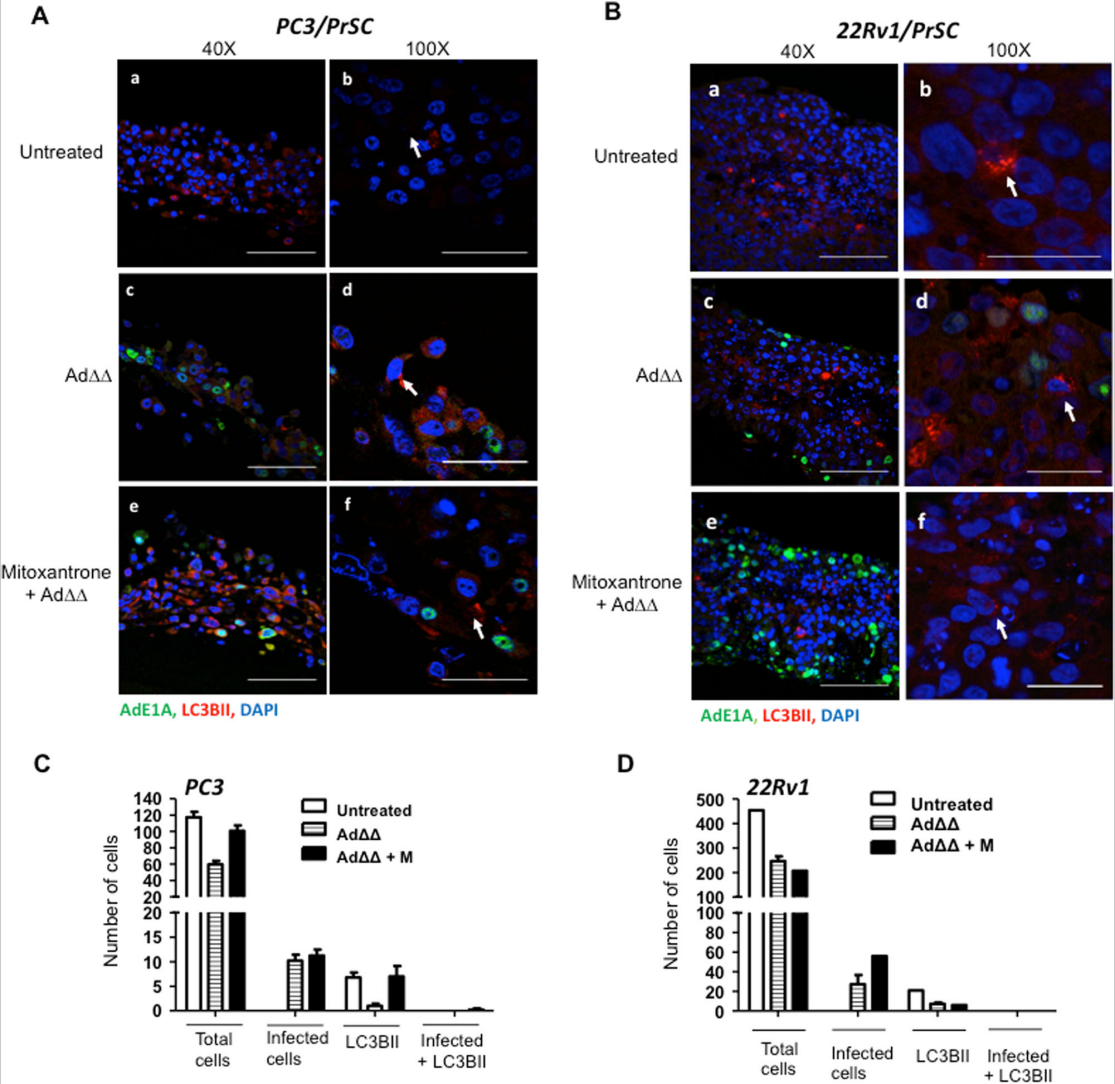
AdΔΔ-infected cells do not express LC3BII in 3-dimenasional co-cultures of PrSC and PC3 or 22Rv1 cells. **a** PC3 and PrSC cells seeded on top of collagen/matrigel matrices and fixed 8 days after seeding, stained for the early viral E1A and cellular LC3BII proteins and nuclei visualised by DAPI using confocal microscopy. **a**,** b** Untreated control. **c**,** d** Cells infected on days 3 and 5 with AdΔΔ (1000 ppc). **e**,** f** Cells treated on days 3 and 5 with mitoxantrone (450 nm) and infected with AdΔΔ. Scale bars: ×40 = 100 µm and ×100 = 50 µm. **b** 22Rv1 and PrSC cells seeded on top of collagen/matrigel matrices and fixed 10 days after seeding. Staining as in **a**. a-b. Untreated control. c−d. Cells infected on days 2 and 6 with AdΔΔ (100 ppc). e-f. Cells treated on days 2 and 6 with mitoxantrone (25 nm) and infected with AdΔΔ. Scale bars: ×40 = 100 µm and ×100 = 25 µm. **a**,** b** White arrows indicate LC3BII-positive cells. All sections stained with AdE1A (green), LC3II (red) and nuclei (blue). **c**,**d** Histograms of quantification of the total number of cells and cells positive for E1A or LC3BII staining per section at ×40 (**a**: PC3 and **b:** 22Rv1), *n* = 1 with 3–4 sections quantified/per condition

**Fig. 6:**
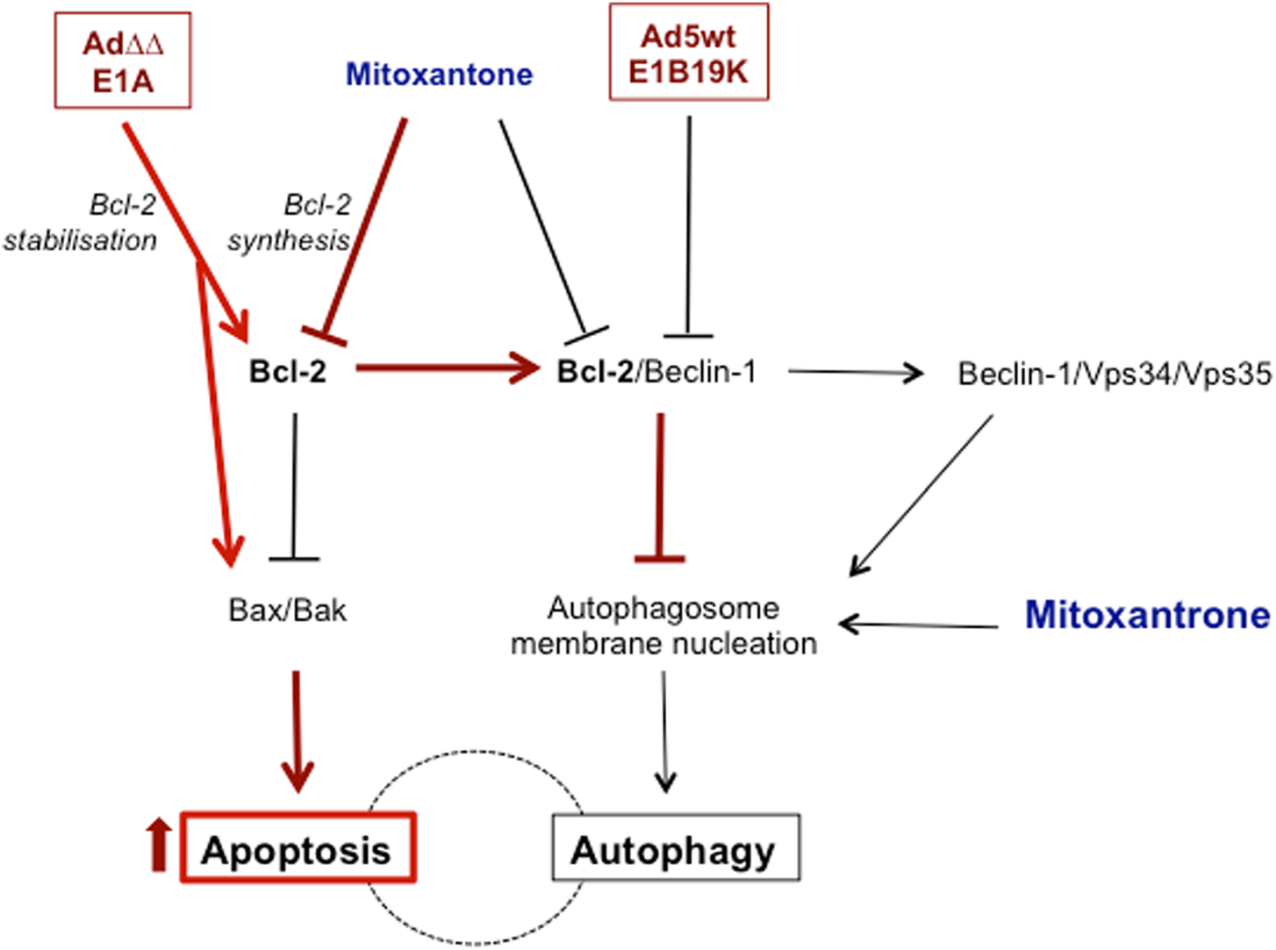
Schematic illustration of activated pathways that play a role in the Ad∆∆ and E1A12S enhancement of mitoxantrone-induced cell killing, highlighting the central role for Bcl-2 in the increased apoptosis. DNA damage caused by the topoisomerase inhibitor mitoxantrone causes cellular cytotoxic stress that activate autophagy as a rescue mechanism. At high levels of cell damage, the cell will be directed towards apoptosis. Mitoxantrone decreases Bcl-2 synthesis that in turn can activate both apoptosis and autophagy dependent on conditions. Mitoxantrone might also have direct effects on the autophagosome membrane nucleation. The viral E1A protein induces an apoptotic response that is prevented by the E1B proteins and may in certain cell lines also activate autophagy that is counteracted by other viral proteins (e.g. E4, protein VI, RID-α). E1A can also stabilise the Bcl-2 protein and decrease autophagy activation. The E1B19K protein is a Bcl-2 homologue and can displace Bcl-2 from Beclin-1 promoting LC3I to II conversion and autophagy induction. In viruses with E1B19K deleted, Ad∆∆ and AdE1A12S, the combination of virus and mitoxantrone shifts the balance towards apoptosis, reducing the survival function of autophagy to increase overall cell killing (indicated by red arrows and lines). Ad5wt expresses E1B19K that activates the Beclin-1/Vps34/Vps35 complex promoting autophagosome formation and does not synergise with mitoxantrone. In Bcl-2 knockdown cells both autophagy and apoptosis are increased

To determine whether the effects of Ad∆∆ on autophagy attenuation occurred in infected cells and/or in surrounding cells through bystander mechanisms, E1A and LC3BII expression were investigated in individual cells. PC3 and 22Rv1 cells were co-cultured with normal prostate stromal (PrSC) cells in 3-dimensional models to mimic the tumour microenvironment in situ. Low levels of LC3BII punctate staining was detected in untreated, Ad∆∆-infected and mitoxantrone-treated cultures after 3–7 days (Fig. 5a, b). Importantly, when cells were co-stained for the early E1A protein, a marker for Ad∆∆-infection, cells were either positive for E1A or LC3BII but not both (Fig. 5a, b; middle and lower panels). These data suggest that conversion of LC3BI to II was attenuated by AdΔΔ in infected cells, but not in surrounding non-infected cells, pointing towards viral factors, mainly E1A, directly altering the response to mitoxantrone (Fig. 4c, d). Taken together, virus and drug may directly influence the balance between apoptosis and autophagy with Bcl-2 playing a central role (Fig. 6).

## Discussion

A promising strategy to overcome treatment-resistance in aggressive cancers is to combine cytotoxic drugs with replication-selective oncolytic adenoviruses. Several clinical and preclinical studies have proved the feasibility of this concept, although the mechanisms for the re-sensitisation are mostly unknown^4,8,14,15,30–32^. We previously demonstrated potent synergistic cell killing with our selective oncolytic mutant Ad∆∆ in combination with mitoxantrone in both cell and in vivo models. Here, we provide evidence that the synergy is dependent on functional Bcl-2 expression. Specifically, viral E1A-expression sensitised the cells to mitoxantrone-mediated apoptosis and simultaneously attenuated mitoxontrone-induced autophagy in prostate cancer cells, only in the presence of Bcl-2 (Fig. 6).

Anti-apoptotic Bcl-2 is frequently upregulated in prostate cancer following androgen ablation therapy^33,34^. Androgens negatively regulate the expression of Bcl-2 by promoting cell cycle progression, releasing E2F from pRb followed by E2F-binding to the Bcl-2 promoter^35^. PC3, PC3M and 22Rv1 express Bcl-2, with high levels in the androgen receptor-negative and drug-insensitive PC3 cells^36^. In line with these reports we observed the most potent synergistic cell killing with Ad∆∆ and mitoxantrone in PC3 and PC3M cells, while sensitisation was lesser in the androgen receptor-positive 22Rv1 cells. We did not determine the absolute levels of Bcl-2, and it is possible that the protein is less abundant and/or active in the 22Rv1 cells due to post-translational regulation by androgens, as reported in patient samples from androgen-sensitive tumours^33^. Specific gene alterations may also influence the activity of Bcl-2. For example, PC3 and PC3M do not express functional p53 and PTEN in addition to the lack of androgen-receptor, while these functions are intact in the drug-sensitive 22Rv1 cells that more readily undergo apoptosis^36–38^.

The Bcl-2 proteins play important roles in the crosstalk between apoptosis and autophagy^39^. Bcl-2 is anti-apoptotic when bound to Bak and prevents Beclin-1 from interacting with the Vps34/Vps35 complex thereby blocking autophagy-initiation^40^. The interactions between adenoviral proteins, autophagy and apoptosis are complex and the interplay has not been clearly defined. The adenoviral E1B19K protein was reported to interact with Beclin-1, disrupting the inhibitory Beclin-1/Bcl-2 complex and promoting formation of the Beclin-1/Vps34/Vps35 complex that initiates autophagy^10^. Viruses with intact E1B19K expression (Ad5wt and Ad∆24) were shown to increase LC3BI to LC3BII conversion in A549 and U87MG cells but not mutants deleted in E1B19K or the entire E1B domain^3,10,41^. Furthermore, AdΔ24 (expressing E1B19K) was demonstrated to induce phosphorylation of Bcl-2 and its dissociation from the Beclin-1/Bcl-2 complex, further supporting autophagy activation^42^. Importantly, we demonstrated that viral mutants deleted in E1B19K, but not intact Ad5wt, counteracted mitoxantrone-stimulated autophagy in prostate cancer cells. Our findings support a role for E1B19K in autophagy-regulation based on the attenuation of drug-dependent autophagy by Ad∆∆ and AdE1A12S, while Ad5wt did not inhibit autophagy or increase apoptosis under our experimental conditions. The E1B19K protein in Ad5wt likely replaced Bcl-2 at Beclin-1 thereby relieving autophagy-inhibition, allowing formation of the activating Beclin-1/Vps34/Vps35 complex. In contrast, mutants deleted in E1B19K (Ad∆∆ and AdE1A12S) express high levels of E1A that promote mitochondrial depolarisation by acting upstream of Bax/Bak and leave the Bcl-2/Beclin-1 inhibitory complex intact^9,11^.

Our findings suggest that mitoxantrone-induced autophagy served as a cellular rescue response to drug-mediated cytotoxicity at suboptimal doses, while at higher drug concentrations (>EC_50_) the pro-survival functions were negated and cells were directed towards apoptotic death. A role for autophagy in the development of treatment-resistance has been suggested although the function of this pathway in prostate cancer is currently unclear^23,43^. In PC3 cells, it was demonstrated that prevention of autophagosome-formation by 3-methyl adenine (3-MA) or Atg5 knockdown promoted apoptosis in response to the anti-tumour drug ursolic acid^28^. Furthermore, combinations of the Akt inhibitor AZD5363 with both early and late autophagy inhibitors, 3-MA, Atg7 siRNA, chloroquine and bafilomycin, caused increased apoptosis in PC3 cells^44^. Taken together, our results with chloroquine, rapamycin, and knockdown of Bcl-2 and Atg7 are in agreement with these reports and point towards autophagy as a protective mechanism in response to drug-induced cytotoxic stress.

We demonstrated that both early and late-stage inhibition of autophagy enhanced mitoxantrone-induced apoptotic cell killing. Knockdown of Atg7 prevented autophagy-initiation and increased apoptosis but did not mimic the viral sensitisation of cells to mitoxantrone at the early 48–72 h time points. In contrast, the late-stage inhibitor chloroquine increased mitoxantrone-mediated cell killing, similar to Ad∆∆-infection. However, based on the decreased LC3II/I ratios in response to viral E1A12S expression, the attenuation of autophagy by Ad∆∆ is likely to occur at an early stage in prostate cancer cells, prior to autophagosome formation in line with the identification of Bcl-2 inhibition of autophagy as an essential requirement for the synergistic cell killing. The high E1A levels as a consequence of the E1B19K-deletion^11^ are likely to stabilise Bcl-2 during the early stages of infection and promote completion of viral protein and DNA synthesis prior to cell lysis. Bcl-2 was reported to facilitate expression of viral proteins, supporting efficient replication^45,46^. Viral proteins including E1A have evolved to support propagation of the virus under extreme conditions such as cytotoxic stress and DNA damage by interfering with multiple cellular processes. Autophagy modulation may be one of these mechanisms that in addition contributes to the synergistic cell killing with apoptosis-inducing drugs.

To conclude, active repression of autophagy by the Bcl-2/Beclin-1 complex is required for Ad∆∆, through E1A-expression, to attenuate drug-induced autophagy and synergistically increase apoptotic cell killing. Our findings support the notion that the viral Bcl-2 homologue E1B19K should be deleted in oncolytic adenoviruses to attain maximal efficacy in combination with current clinical therapies and that expression of Bcl-2 supports the propagation of these mutants in tumour cells. The frequent overexpression of Bcl-2 in the majority of cancers including prostate cancer^25^ provides an excellent opportunity to treat these tumours with Ad∆∆ in combination with apoptosis-inducing drugs. In light of our studies and reports from clinical trials with oncolytic adenoviruses, we expect that future clinical applications of viral mutants will be efficacious in combination with current clinical cytotoxic drugs in Bcl-2-positive cancers. In addition, our findings demonstrate that for optimal synergy between the oncolytic mutants and drugs, attenuation of autophagy by the Bcl-2/Beclin-1 complex need to be preserved.

## Material and methods

### Cell lines and culture conditions

The human prostate carcinoma cell lines 22Rv1 (ATCC, VA), PC3 (ECACC, UK), the more aggressive PC3 subline PC3M^47^ (Dr. Rajan, BCI, London, UK) and the human embryonic kidney cell line HEK293 (Cancer Research UK Cell Services) were cultured in Dulbecco’s modified Eagle’s medium (DMEM) containing 1% penicillin and streptomycin (P/S) and 10% fetal bovine serum (FBS) (Sigma-Aldrich) at 37 °C and 5% CO_2_. Cell lines were authenticated by short tandem repeat profiling (LGC Standards, UK) and verified to be identical to the profiles reported by the suppliers and to the original vial.

### Viruses and infections

Ad5wt and Ad∆∆ viruses were previously generated from species C adenovirus type 5 plasmids, produced, purified and characterised as previously described^7,8^. Ad∆∆ is replication-selective (E1ACR2- and E1B19K-deleted). The non-replicating AdE1A12S and AdGFP express only E1A12S or GFP constitutively from the CMV-promoter replacing the E1-region^15^. The replicating viruses had viral particle (vp)/infectious unit (pfu) ratios of 5–15 vp/pfu. All infections were performed in serum-free DMEM for 2 h and replaced with 10% FBS/1% P/S DMEM ± indicated doses of drug(s).

### Cell viability and death assays

Dose−response curves to mitoxantrone (Baxter, UK) were generated by serial dilutions to determine concentrations killing 50% of cells (EC_50_). Cell viability was analysed 3–5 days after treatment using the 3-(4,5-dimethylthiazol-2-yl)-5-(3-carboxymethoxyphenyl)-2-(4-sulfophenyl)-2H-tetrazolium assay (MTS; Promega) to quantify live cells as an indirect measurement of cell death^11^. Fixed doses of viral mutants, rapamycin (Calbiochem, CA) or chloroquine (Sigma-Aldrich), that killed <40% of cells alone were added to the dose−response curves. Data were presented as percentages of the EC_50_-values after correction for cell death induced by the corresponding controls (virus, inhibitor, drug or cells alone).

Synergistic interactions were determined at three constant dilution ratios of viruses and mitoxantrone at 0.5, 2.5 and 12.5 viral particles per cell (ppc)/nm drug, and isobolograms were generated from individual EC_50_-values followed by determination of combination index (CI) as previously described^15^.

Direct measurements of cell killing efficacy was by the Trypan blue inclusion assay using a TC20^TM^ automated cell counter (Bio-Rad, CA), as previously described^11^.

### Immunoblotting

Cells were infected and/or treated and cell lysates prepared after 48 h, in RIPA buffer (50 mm Tris-HCl, 150 mm NaCl, 1 mm EDTA, 1% (v/v) NP40 and 0.1% (w/v) SDS) containing phosphatase and protease inhibitors (PhosphoSTOP; Roche Diagnostics, Switzerland). Proteins were quantified using the Bio-Rad Protein assay (Bio-Rad), prepared in sample Laemmli buffer (0.125 m Tris-HCl pH6.8, 20% glycerol, 4% SDS, 0.01% bromophenol blue, 10% β-mercaptoethanol) and 15–20 μg of total protein were separated by SDS-polyacrylamide gel electrophoresis (SDS-PAGE) under reducing conditions, transferred to polyvinylidene difluoride membranes (Merck-Millipore, MA) and identified with the following antibodies: rabbit anti-LC3B (1:1000), goat anti-Ad hexon (1:1000) and mouse anti-vinculin (1:5000) (Abcam, UK), mouse anti-Bcl-2 (1:1000; Dako, UK), mouse anti-AdE1A (1:2000; GeneTex, TX), rabbit anti-Atg5, rabbit anti-PARP and mouse anti-p62 (1:1000; Santa Cruz Biotechnology, CA) and goat anti-actin, goat anti-Ku-70 (1:5000; Santa Cruz Biotechnology) and rabbit anti-Atg7 (1:2000; Merck-Millipore). Detection was by horseradish peroxidase-conjugated secondary antibodies (Dako) and the enhanced chemiluminescence reagent Plus-ECL (PerkinElmer, MA), visualised by autoradiography X-Ray film or digital capture (Chemidoc; GE Healthcare, UK). Densitometric analysis was performed using the NIH ImageJ software.

### 3-dimensional cultures, immunostaining and confocal microscopy

Normal prostate stromal PrSC (Lonza, MD) and PC3 or 22Rv1 cells were mixed at ratios of 1:1–3:1, and a total of 100,000 cells were seeded on top of collagen type I-Matrigel matrix (Corning, UK) in transwells as previously described for pancreatic and breast cancer cultures^48,49^. The 3-dimensional cultures were infected with Ad∆∆ and treated with mitoxantrone (100 ppc, 25 nm; PrSC-22Rv1 and 1000 ppc, 450 nm; PrSC-PC3) at indicated time points and cultured for 8–10 days, fixed in formalin, paraffin embedded and sectioned (4 µm/section). Immunostaining was performed by the following antibodies: rabbit anti-LC3B (1:500; Abcam), mouse anti-AdE1A (1:2000; GeneTex), goat Alexa Fluor anti-mouse M488 (green), chicken Alexa Fluor anti-rabbit R594 (red) (ThermoFisher, UK) and nuclei was stained by 4′,6-diamidino-2-phenylindole (DAPI, blue; Sigma-Aldrich). Images were acquired by confocal laser-scanning microscopy (Zeiss LSM710) at x40–x100 with 405, 488 and 543 nm lasers. Positive cells were quantified manually using the NIH ImageJ software. Total number of cells positive for DAPI, LC3B and E1A were counted in each section and at least 4 sections/sample were analysed at the same magnification.

### Viral replication assay

Cells were infected with 100 ppc, media and cells collected after 48–72 h, freeze-thawed and assayed in HEK293 cells by the tissue culture inhibitory dose at 50% (TCID_50_) as previously described^8^. Each sample was determined in triplicate and data from >2 studies were averaged and expressed as pfu/cell ± SD.

### Viral genome amplification by qPCR

Cells were infected with 25–3000 ppc and/or treated with 50 nm rapamycin, 10 μm chloroquine and/or mitoxantrone at 25–900 nm. DNA was extracted using the QIAamp DNA Blood Mini Kit (Qiagen, UK) after 2, 48 and 72 h, and 10 ng were amplified and quantified as previously described using the viral E2A gene, Power SYBR Green Master Mix and the 7500 Real Time PCR System (Applied Biosystems, CA)^13^. Results were expressed as the ratio of viral genome copies at each time point relative to that at 2 h after infection.

### Reverse transcription (RT)-qPCR

PC3 cells were infected with 500 ppc of Ad5wt or AdΔΔ ± mitoxantrone 450 nm for 12 and 48 h, RNA was extracted by the RNeasy Mini Kit (Qiagen) and 0.7 μg of purified mRNA were transcribed into cDNA (High-Capacity RNA-to-cDNA™ Kit; Applied Biosystems). The qPCR was performed as described above using the following primers: Bcl-2 5′-GCCCTGTGGATGACTGAGTA-3′ (forward) and 5′-GCCAGGAGAAATCAAACAGAGG-3′ (reverse), GAPDH 5′-TGGGCTACACTGAGCACCAG-3′ (forward) and 5′-GGGTGTCGCTGTTGAAGTCA-3′ (reverse). Bcl-2 values were normalised to GAPDH and presented as the difference compared to the average ratios of the 12 h time point in each sample.

### Flow cytometry for Annexin V and viral infection

PC3 cells were infected with 500 ppc AdΔΔ ± mitoxantrone 450 nm, harvested, resuspended in Annexin-binding buffer (10 mm HEPES, 140 mm NaCl, and 2.5 mm CaCl_2_, pH7.4) and incubated with Alexa Fluor^®^ 488 Annexin V (1:50; Invitrogen, CA) and DAPI 120 h after treatment. Detection was by BD LSRFortessa™ gated with lasers 585/30 nm vs. 450/50 nm and analysed by the BD FACSDiva™ software (Becton Dickinson, UK) after acquisition of 10,000 events. Cells were infected with AdGFP (10–1000 ppc) ± mitoxantrone (25–900 nm), ±rapamycin (50 nm) or chloroquine (5–10 μm), harvested 48 h later and GFP-expression detected by a FACS Calibur cytometer (Becton Dickinson) and analysed as described above.

### Mitochondrial depolarisation and acidic vesicle formation

Cells were infected with AdΔΔ ± mitoxantrone (20–500 ppc ± 10–900 nm), ±rapamycin (50 nm), or chloroquine (10 μm) for 24, 48, 72, 96 and 120 h. Attached and floating cells were collected, stained with 60 ng/ml tetramethylrhodamine ethyl ester perchlorate (TMRE; Molecular Probes/Invitrogen) in PBS containing DAPI (1 µg/ml) and analysed on the LSRFortessa™ with lasers YG585/15 nm vs. V450/50 nm, and apoptosis quantified using the BD FACSDiva™ software (10,000 events). Overnight treatment with staurosporine (1 μm; Sigma-Aldrich) was included as a positive control.

Acidic vesicle formation was determined by infection with AdΔ∆ ± mitoxantrone (20–500 ppc ± 450 nm) for 72–120 h. Lysotracker^®^ green (50 nm; Invitrogen) was added for 1 h, and formation of acidic vesicles quantified by the 530 nm laser (LSRFortessa™) and the FACSDiva™ software (10,000 events).

### siRNA transfections

PC3 cells were seeded in six-well plates (2×10^5^cells/well) and transfected with 50 nm of siGENOME non-targeting (NT) siRNA#2 (D-001206-14-05), and the following onTARGET plus SMARTpools; Bcl-2 siRNA (L-003307-00), Atg5 siRNA (L-004374-00) or Atg7 siRNA (L-020112-00), using DharmaFECT 2 transfection reagent (Dharmacon) according to the manufacturer’s instructions. Cells were harvested after 18–24 h, counted and re-seeded in 96-well plates for cell viability studies, immunoblotting and apoptosis (TMRE) flow cytometry.

## Electronic supplementary material

Supplementary Figures legends

Supplementary Figures
