# Supplementary material for: Sensitisation to mitoxantrone-induced apoptosis by the oncolytic adenovirus Ad∆∆ through Bcl-2-dependent attenuation of autophagy
Source: Oncogenesis. 2018 Jan 24;7(1):6. doi: 10.1038/s41389-017-0020-8 (PMC5833340; doi:10.1038/s41389-017-0020-8)
Supplement: Supplementary file 1 — Supplementary Figures legends [file 41389_2017_20_MOESM1_ESM.docx]

**Supplementary Figure legends**

**Supplementary Figure 1. AdΔΔ increases mitoxantrone–induced apoptotic cell killing in PC3M and 22Rv1 cells.** **A.** PC3M cells treated with mitoxantrone (450 and 900nM) and/or infected with AdΔΔ (20ppc). Cell death determined with the MTS assay 5 days after treatment. One-way Anova with Tukey-Kramer, averages, SEM, ****p<0.0001, compared to the theoretical additive value of mitoxantrone and virus, n=2. **B.** 22Rv1 cells analysed at the indicated time points for mitochondrial depolarization by loss of TMRE staining using flow cytometry, after infection with AdΔΔ at 20ppc and/or treated with mitoxantrone at 10nM, n=2 **C.** PC3M cells treated with mitoxantrone (450 and 900nM) and/or Ad5wt or AdΔΔ (20ppc) and analysed 3 days after treatment for mitochondrial depolarisation by loss of TMRE staining, one-way Anova with Tukey-Kramer post-test, ***p<0.001, ****p<0.0001, n=3. **D**. The viral E1A protein expressed after infection with AdΔΔ or AdE1A12S in the presence of mitoxantrone in PC3 cells. Infection with AdΔΔ or AdE1A12S (Ad12S) (500ppc) and/or treated with mitoxantrone (500nM) and lysed 24 or 48h after treatment. E1A expression was assessed by immunoblotting, representative blots, n=3.

**Supplementary Figure 2. Bcl-2 knock-down prevents AdΔΔ-mediated sensitization to mitoxantrone in PC3 cells.** **A.** Percent cell death in PC3 cells transfected with non-targeting siRNA (siNT), Bcl-2 siRNA or the transfection reagent alone (Mock) determined by the MTS assay. Mitoxantrone (450nM), AdΔΔ (500ppc) and the combinations, n=4. **B.** Percent cell death in PC3 cells, n=3. A-B. No significant changes by One-way Anova with Tukey-Kramer post-test.

**Supplementary Figure 3. AdΔΔ attenuates mitoxantrone–induced autopahgy in prostate cancer cell lines.**  **A.** Expression of LC3BII/I and p62 by immunoblotting in PC3M cells treated with mitoxantrone (450 and 900nM) and/or AdΔΔ (20ppc) and analysed 2 days after treatment. One representative blot, n=2. Right panel; Ratios of LC3BII/I and p62 levels were quantified by densitometer, normalised to the loading control and expressed as fold-change relative to the untreated control. **B.** LC3II/I ratios from immunoblotting data and apoptosis determined by loss of TMRE staining (72h) in PC3M cells. In all assays cells were treated with mitoxantrone at 450nM and/or infected with AdΔΔ at 20ppc, n=2-3.

**Supplementary Figure 4. Rapamycin promotes and chloroquine blocks autophagy in PC3 and 22Rv1 cells**. **A-B.** Expression of p62 and LC3BII/I in PC3 (A) and 22rv1 (B) cells treated with chloroquine (CQ) or rapamycin (R) at indicated doses and analysed at 24, 48 and 72h after treatment, representative immunoblots, n=2. **C.** Viral infection in the presence of mitoxantrone and autophagy modulators. PC3 cells were treated with mitoxantrone (M;900nM) and/or CQ (10μM), or rapamycin (50nM) and 22Rv1 cells with mitoxantrone (M;25nM) and/or CQ (10μM), or rapamycin (R; 50nM) for 24h followed by AdGFP-infection (PC3;1000ppc and 22Rv1;10ppc) and analysed for GFP-expression by flow cytometry after 48h, one-way Anova with Tukey-Kramer post-test, **p<0.001, ***p<0.001, n=3. **D.** Representative immunoblots of E1A expression in PC3 and 22Rv1 cells treated with mitoxantrone and/or autophagy modulators. PC3 cells were infected with AdΔΔ (500ppc) and/or treated with mitoxantrone (M;450nM) and/or rapamycin (R;50nM) and/or CQ (10μM) and 22Rv1 cells were infected with AdΔΔ (20ppc) and/or treated with mitoxantrone at (M;10nM) and/or rapamycin R;50nM) and/or chloroquine (CQ;10μM), analysed after 48h. **E.** Viral replication determined by TCID_50_. Cells were infected with AdΔΔ (PC3;200ppc and 22Rv;100ppc) and treated with mitoxantrone (PC3;900nM and 22Rv1;25nM), rapamycin (R; 50nM) or CQ (10μM) for 72h, n≥2.

**Supplementary Figure 5. Autophagy inhibition with Atg7 knock-down does not prevent Ad∆∆-mediated sensitisation to mitoxantrone in PC3 cells.** Ratios of EC_50_-values for mitoxantrone in PC3 cells treated with non-targeting siRNA (siNT), Atg5 siRNA (siAtg5) or the transfection reagent alone (Mock). Cells were infected with AdΔΔ (500ppc) and treated with mitoxantrone (450nM) 4 days after transfection and analysed 48h after treatment with the MTS assay. Data expressed as EC_50_ for the combined treatment/EC_50_ mitoxantrone, unpaired t-test, **p<0.01 ****p<0.0001, n=3. Representative immunoblot verifying Atg7 knockdown in each viability study, n≥3.
