# Supplementary material for: Sensitisation to mitoxantrone-induced apoptosis by the oncolytic adenovirus Ad∆∆ through Bcl-2-dependent attenuation of autophagy
Source: Oncogenesis. 2018 Jan 24;7(1):6. doi: 10.1038/s41389-017-0020-8 (PMC5833340; doi:10.1038/s41389-017-0020-8)
Supplement: Supplementary file 2 — Supplementary Figures [file 41389_2017_20_MOESM2_ESM.pptx]

## Slide 1
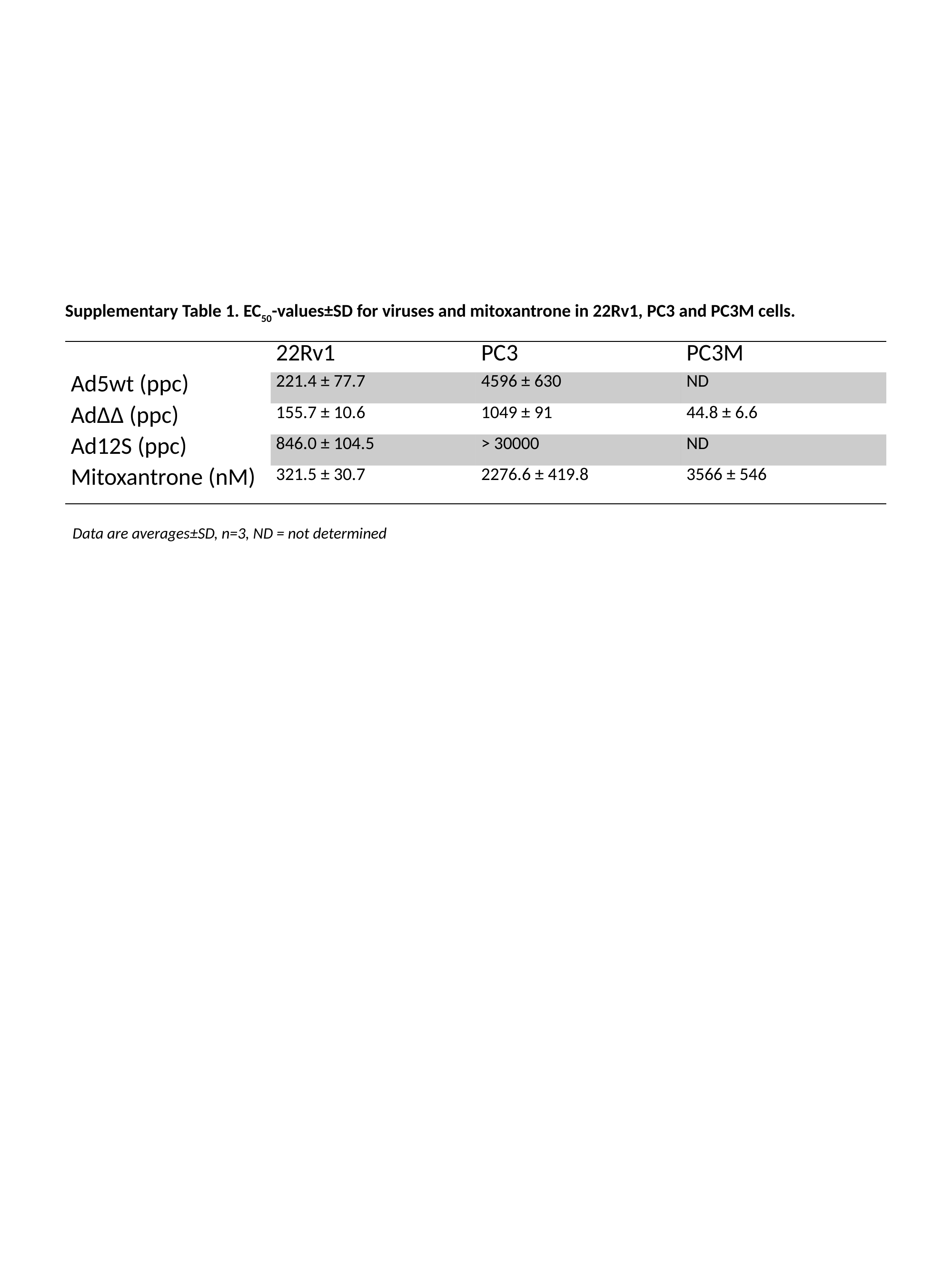

Supplementary Table 1. EC50-values±SD for viruses and mitoxantrone in 22Rv1, PC3 and PC3M cells.
| | 22Rv1 | PC3 | PC3M |
| --- | --- | --- | --- |
| Ad5wt (ppc) | 221.4 ± 77.7 | 4596 ± 630 | ND |
| AdΔΔ (ppc) | 155.7 ± 10.6 | 1049 ± 91 | 44.8 ± 6.6 |
| Ad12S (ppc) | 846.0 ± 104.5 | ˃ 30000 | ND |
| Mitoxantrone (nM) | 321.5 ± 30.7 | 2276.6 ± 419.8 | 3566 ± 546 |
Data are averages±SD, n=3, ND = not determined

## Slide 2
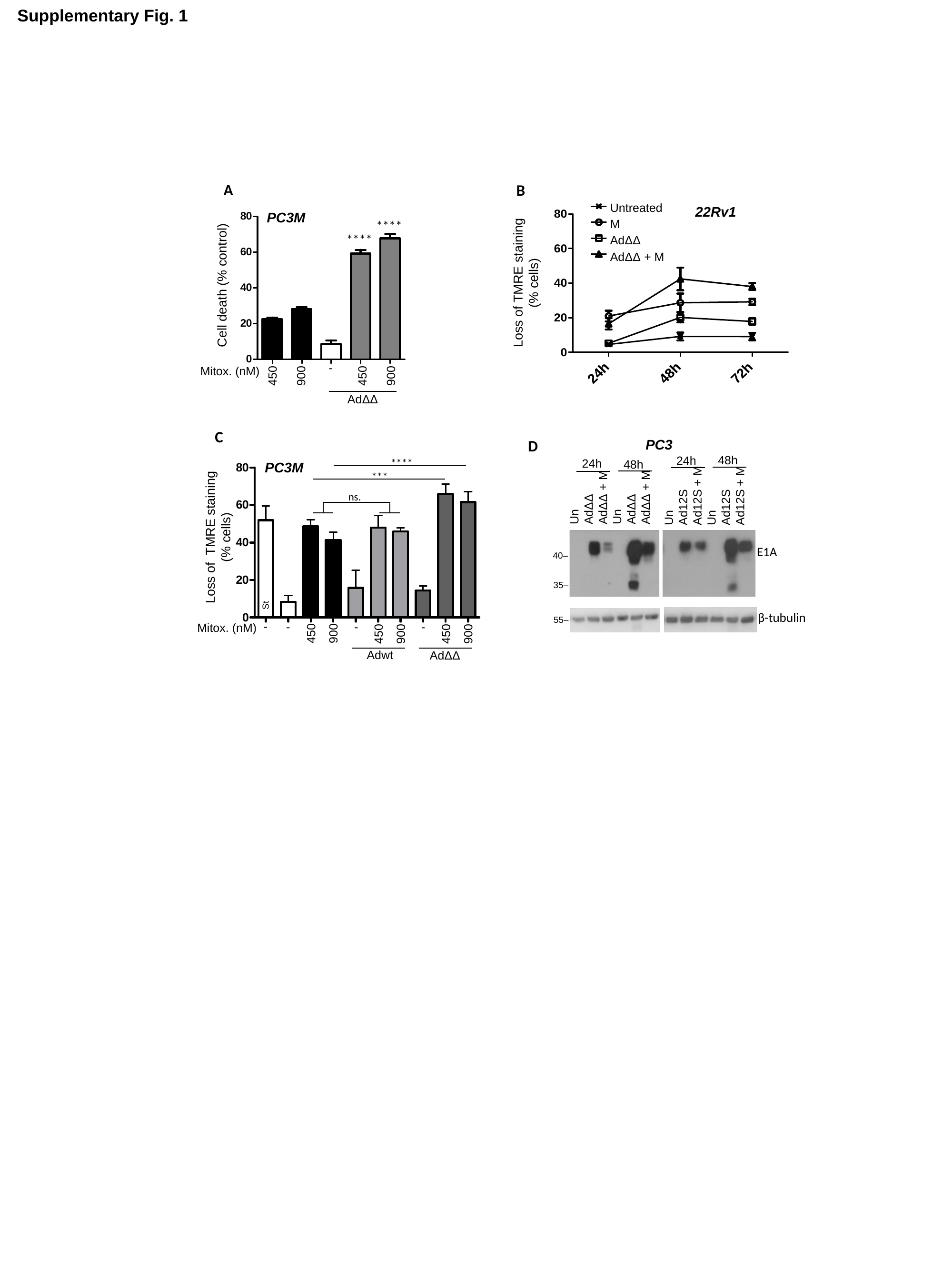

Supplementary Fig. 1
A
****
****
Cell death (% control)
450
900
450
900
-
Mitox. (nM)
AdΔΔ
B
Untreated
M
AdΔΔ
AdΔΔ + M
Loss of TMRE staining
(% cells)
22Rv1
PC3M
C
D
PC3
****
***
ns.
Loss of TMRE staining
(% cells)
900
450
900
450
900
450
-
-
-
-
Mitox. (nM)
Adwt
AdΔΔ
St
48h
24h
24h
Un
AdΔΔ
AdΔΔ + M
Un
AdΔΔ
AdΔΔ + M
48h
Un
Ad12S
Ad12S + M
Un
Ad12S
Ad12S + M
E1A
β-tubulin
40–
35–
55–
PC3M

## Slide 3
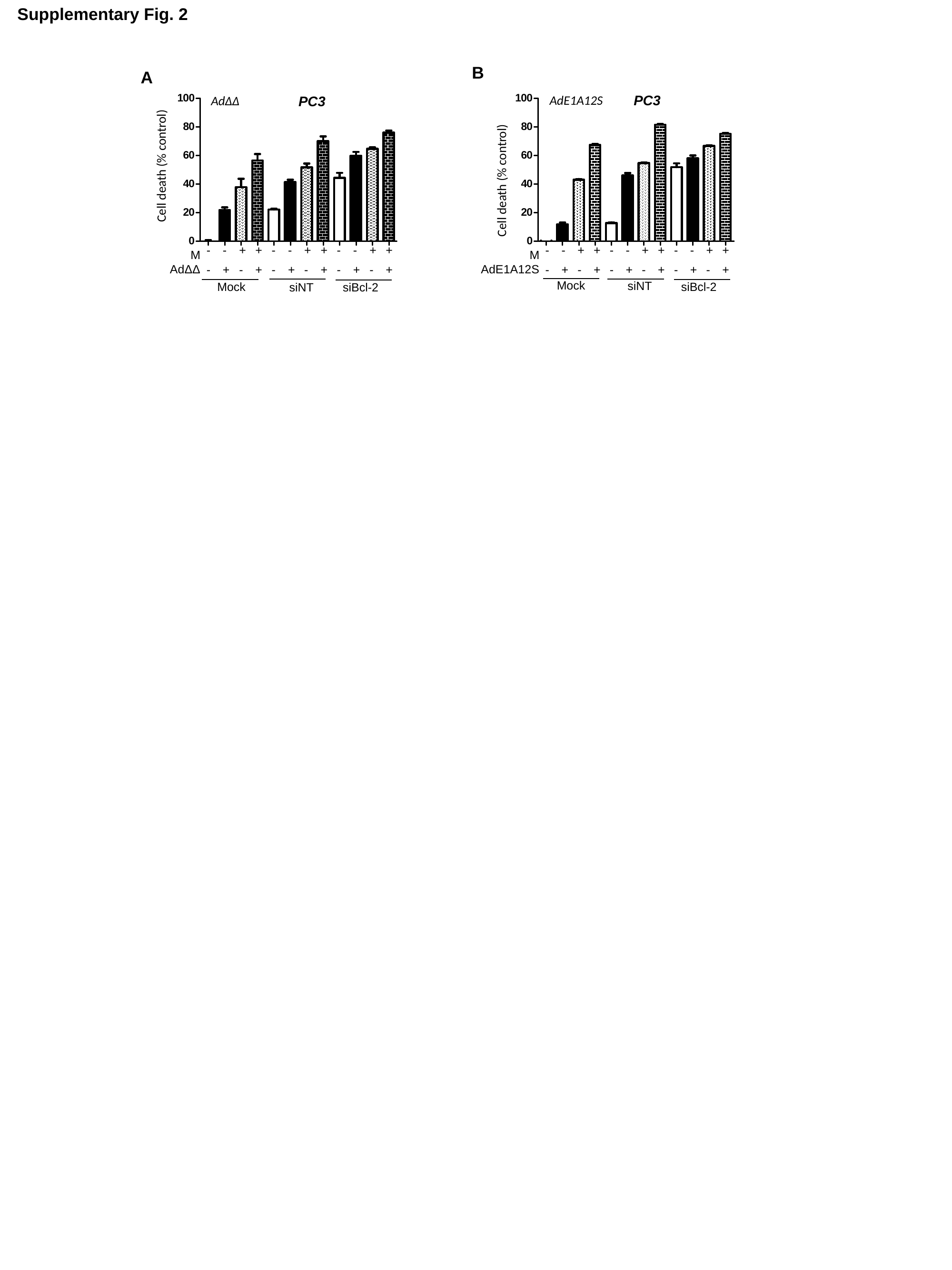

Supplementary Fig. 2
B
A
PC3
AdE1A12S
AdΔΔ
PC3
Cell death (% control)
Cell death (% control)
| - | - | + | + | - | - | + | + | - | - | + | + |
| --- | --- | --- | --- | --- | --- | --- | --- | --- | --- | --- | --- |
| - | + | - | + | - | + | - | + | - | + | - | + |
| - | - | + | + | - | - | + | + | - | - | + | + |
| --- | --- | --- | --- | --- | --- | --- | --- | --- | --- | --- | --- |
| - | + | - | + | - | + | - | + | - | + | - | + |
M
AdE1A12S
M
AdΔΔ
Mock
siNT
Mock
siBcl-2
siBcl-2
siNT

## Slide 4
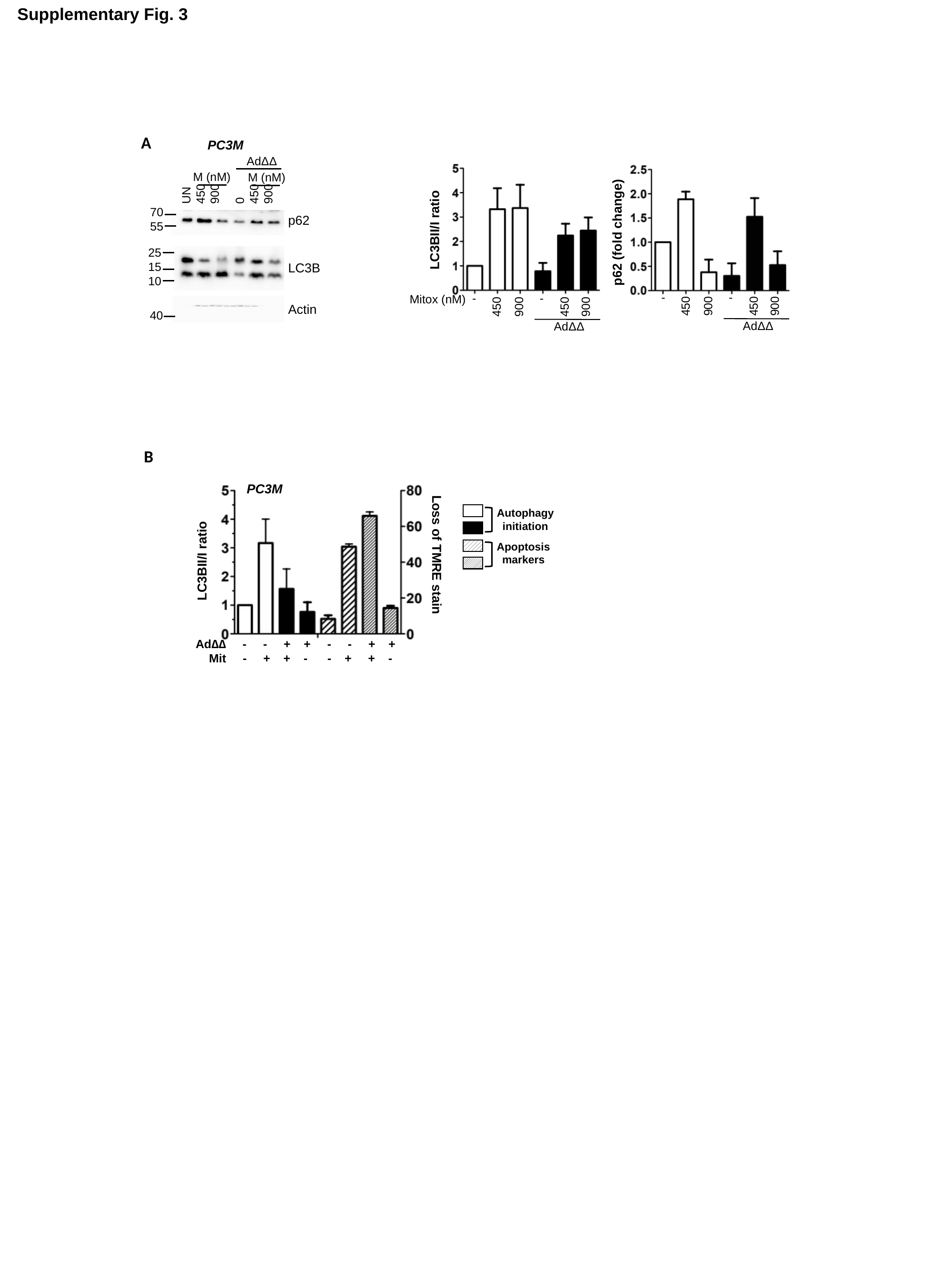

Supplementary Fig. 3
A
PC3M
AdΔΔ
 M (nM)
UN
450
900
0
450
900
 M (nM)
70
55
p62
LC3B
Actin
25
15
10
40
LC3BII/I ratio
-
-
Mitox (nM)
450
900
900
450
AdΔΔ
p62 (fold change)
-
-
450
900
900
450
AdΔΔ
B
PC3M
Autophagy
initiation
Apoptosis
markers
LC3BII/I ratio
Loss of TMRE stain
 Ad∆∆ - - + + - - + +
 Mit - + + - - + + -

## Slide 5
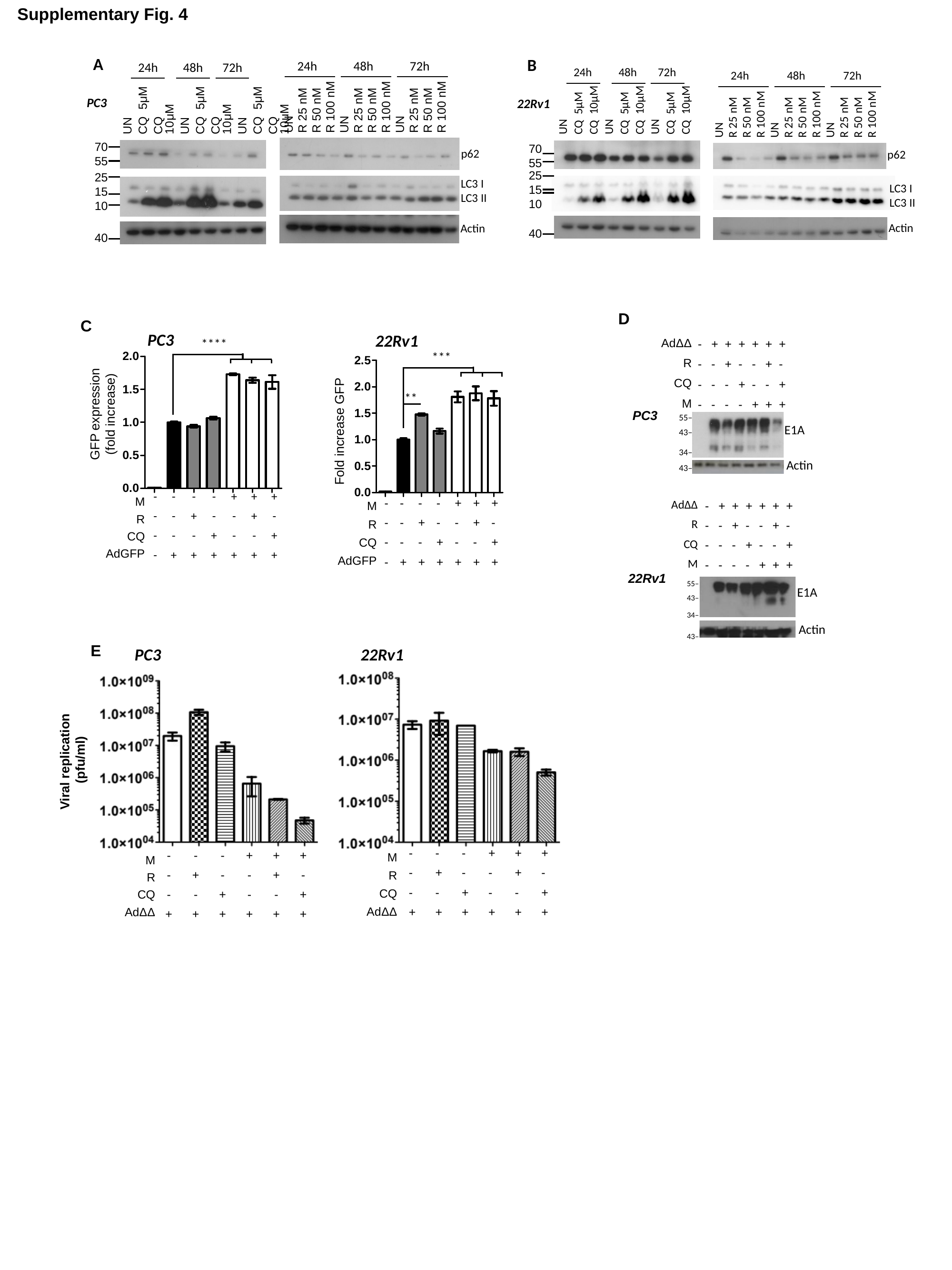

Supplementary Fig. 4
UN
R 25 nM
R 50 nM
R 100 nM
UN
R 25 nM
R 50 nM
R 100 nM
UN
R 25 nM
R 50 nM
R 100 nM
24h
48h
72h
LC3 I
LC3 II
A
24h
48h
72h
UN
CQ 5μM
CQ 10μM
UN
CQ 5μM
CQ 10μM
UN
CQ 5μM
CQ 10μM
70
55
p62
25
15
10
Actin
40
B
UN
R 25 nM
R 50 nM
R 100 nM
UN
R 25 nM
R 50 nM
R 100 nM
UN
R 25 nM
R 50 nM
R 100 nM
24h
48h
72h
LC3 I
LC3 II
24h
48h
72h
UN
CQ 5μM
CQ 10μM
UN
CQ 5μM
CQ 10μM
UN
CQ 5μM
CQ 10μM
70
55
p62
25
15
10
Actin
40
PC3
22Rv1
D
C
PC3
22Rv1
****
| AdΔΔ |
| --- |
| R |
| CQ |
| M |
| - | + | + | + | + | + | + |
| --- | --- | --- | --- | --- | --- | --- |
| - | - | + | - | - | + | - |
| - | - | - | + | - | - | + |
| - | - | - | - | + | + | + |
***
**
GFP expression
(fold increase)
PC3
55–
Fold increase GFP
E1A
43–
34–
Actin
43–
| - | - | - | - | + | + | + |
| --- | --- | --- | --- | --- | --- | --- |
| - | - | + | - | - | + | - |
| - | - | - | + | - | - | + |
| - | + | + | + | + | + | + |
M
R
CQ
AdGFP
| - | - | - | - | + | + | + |
| --- | --- | --- | --- | --- | --- | --- |
| - | - | + | - | - | + | - |
| - | - | - | + | - | - | + |
| - | + | + | + | + | + | + |
M
R
CQ
AdGFP
| - | + | + | + | + | + | + |
| --- | --- | --- | --- | --- | --- | --- |
| - | - | + | - | - | + | - |
| - | - | - | + | - | - | + |
| - | - | - | - | + | + | + |
| AdΔΔ |
| --- |
| R |
| CQ |
| M |
22Rv1
55–
43–
34–
43–
E1A
Actin
E
PC3
22Rv1
Viral replication
(pfu/ml)
| - | - | - | + | + | + |
| --- | --- | --- | --- | --- | --- |
| - | + | - | - | + | - |
| - | - | + | - | - | + |
| + | + | + | + | + | + |
| - | - | - | + | + | + |
| --- | --- | --- | --- | --- | --- |
| - | + | - | - | + | - |
| - | - | + | - | - | + |
| + | + | + | + | + | + |
M
R
CQ
AdΔΔ
M
R
CQ
AdΔΔ

## Slide 6
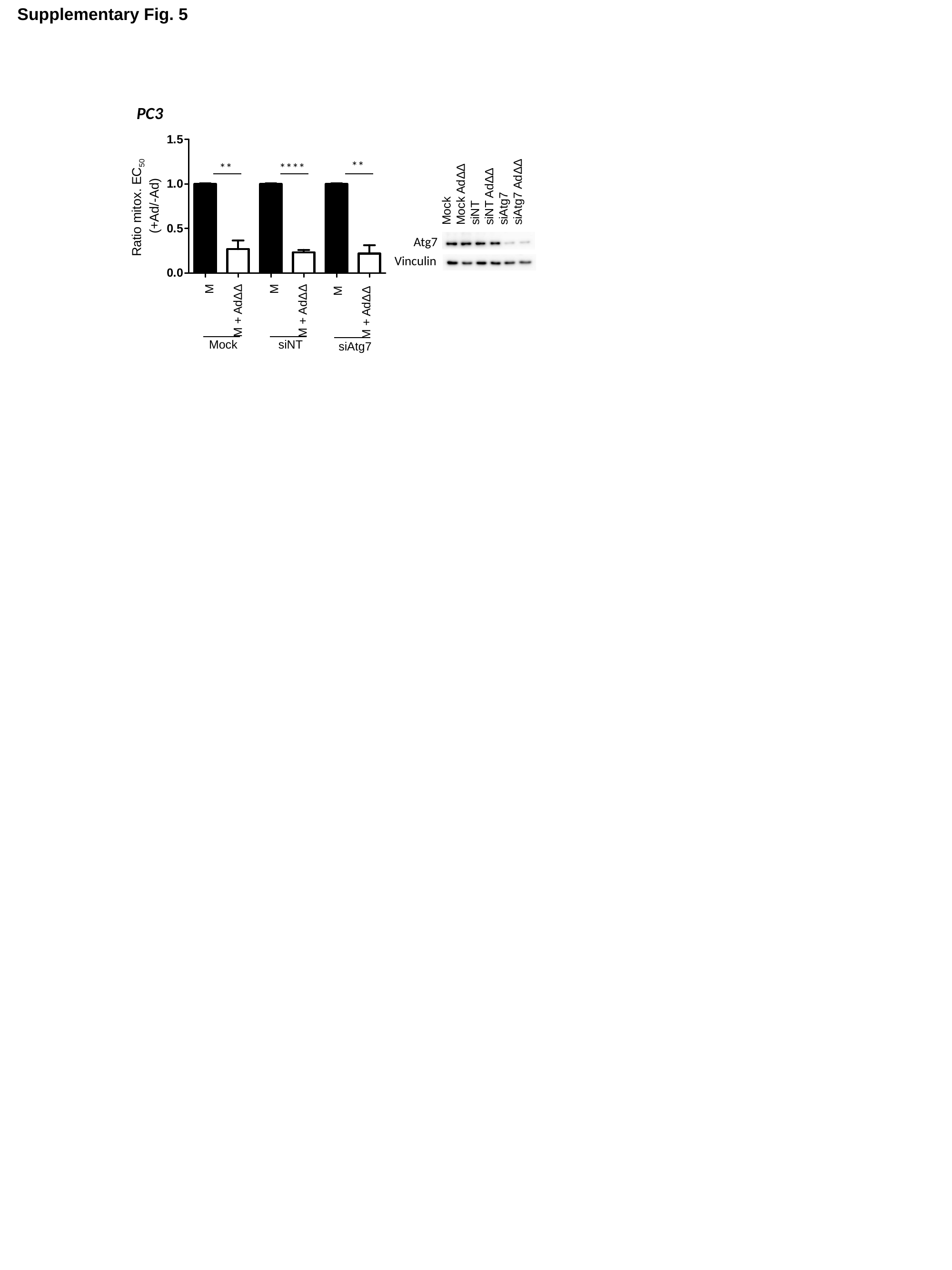

Supplementary Fig. 5
PC3
**
****
**
M
M + AdΔΔ
M
M + AdΔΔ
M
M + AdΔΔ
siNT
Mock
siAtg7
Mock
Mock AdΔΔ
siNT
siNT AdΔΔ
siAtg7
siAtg7 AdΔΔ
Atg7
Vinculin
Ratio mitox. EC50
(+Ad/-Ad)
